# Clinical Characteristics, Etiology, Radiological Features, and Outcomes of Intracerebral Hemorrhage in Young Adults at King Abdulaziz Medical City, Ministry of National Guard Health Affairs, Riyadh, Kingdom of Saudi Arabia

**DOI:** 10.1155/srat/5550380

**Published:** 2025-08-21

**Authors:** Ismail A. Khatri, Moath Almosa, Abdulah Alkahtani, Khaled Alanazi, Nazish Masud

**Affiliations:** ^1^Department of Neurology, King Abdulaziz Medical City, MNGHA, Riyadh, Saudi Arabia; ^2^College of Medicine, King Saud bin Abdulaziz University for Health Sciences, Riyadh, Saudi Arabia; ^3^King Abdullah International Medical Research Center, Riyadh, Saudi Arabia; ^4^King Faisal Specialist Hospital and Research Center, Riyadh, Saudi Arabia; ^5^Department of Medicine, King Fahad Medical City, MOH, Riyadh, Saudi Arabia; ^6^Jiann-Ping Hsu College of Public Health, Department of Epidemiology, Georgia Southern University, Statesboro, Georgia, USA

**Keywords:** intracerebral hemorrhage, outcome, radiological features, Saudi Arabia, young adults

## Abstract

**Background:** Hypertension is the most common cause of intracerebral hemorrhage (ICH). The presentation, etiology, and outcome of ICH among young adults may vary compared to other age groups. The prior literature from our region has described a variety of etiologies with an inconsistent relationship to hypertension, which is the commonest cause of primary ICH in adults overall.

**Objective:** We aimed to determine the demographic pattern, clinical presentation, underlying etiology, radiological characteristics, and outcome of ICH among young adults in our population.

**Methods:** This was an IRB-approved chart review that included patients from January 2016 to December 2020. Descriptive young adults were defined as people between 15 and 45 years and arbitrarily divided into threee further age groups. A variety of demographic, clinical, and radiological features were compared among the subgroups and presented as descriptive and comparative analyses.

**Results:** A total of 120 patients were included; 110 (91.7%) were males. The mean age was 26.8 ± 7.4 years. Majority, 86 (73.5%), presented with loss of consciousness; 22 (18.8%) had seizures, 14 (13.2%) had headaches, and 13 (11.9%) had vomiting. Traditional vascular risk factors, hypertension (5.8%) and diabetes mellitus (2.5%) were uncommon. Mean GCS was 7 ± 4. The commonest cause was trauma in 101 (84.2%) patients. Lobar hemorrhage was the commonest, 99 (83.2%); 92 (81.4%) had ICH volume < 30 mL, and ventricular involvement was seen in 43 (36.1%). Median ICH score was 2. Higher odds of mortality were observed among the oldest age group (OR 4.30, 95% CI 1.23–14.98, *p* = 0.022), higher ICH scores (OR 3.37, 95% CI 1.86–6.09, *p* < 0.001), ICH volume > 30 mL (OR 16.40, 95% CI 5.35–50.26, *p* < 0.001), ventricular extension (OR 5.60, 95% CI 2.14–14.68, *p* < 0.001), and nontraumatic ICH etiology (OR 3.59, 95% CI 1.26–10.26, *p* = 0.017).

**Conclusions:** In our cohort, ICH was more common in young males; trauma being the leading cause of ICH. ICH resulted in significant morbidity and mortality in this population. Larger hemorrhages, ventricular involvement, and relatively older age were poor prognostic factors.

## 1. Background

Intracerebral hemorrhage (ICH) accounts for approximately 10%–15% of all cases of stroke worldwide, carrying a significant risk of permanent disability and mortality [1]. It results from a rupture of blood vessels inside the brain parenchyma and can be classified into primary and secondary [2]. Primary ICH is associated with hypertension and amyloid angiopathy, when diseased intracranial small vessels spontaneously rupture [2, 3]. Secondary ICH occurs in relation to an underlying vascular malformation, vasculitis, coagulopathy, tumors, trauma, or other conditions [2].

The annual incidence of ICH in young adults (defined as aged 18–50 years) is around five per 100,000 individuals [4]. Many studies defined young adults as adults up to the age of 45 years. Multiple, single-center studies had been conducted across the world, reporting variable leading etiologies depending on the chosen upper limit of age, race, and geographical factors. Studies on the Asian population reported hypertension to be the leading cause of ICH in young adults, while western studies showed a high prevalence of vascular malformation in addition to hypertension [5–9].

Only two prior studies from Saudi Arabia evaluated ICH in young; the first was published in 1998 [10]. It was a multicenter study with a total of 107 patients (aged 6–months to 45 years). It reported arteriovenous malformations (AVMs) to be the most common cause (23%) followed by hypertension (20%) and blood disorders (16%). No identifiable cause was found in 26% of their patients. They reported a direct relationship between hypertensive ICH cases with an increase in age, as it represented 6%–8% of the cases in the first two decades and 39% in those aged 40–45 [10]. The second study was a retrospective study of patients aged 18–55 who suffered from hypertensive ICH. They divided the patients into two groups depending on their age (30–45 and 45–55). They concluded that younger patients with ICH had worse clinical outcomes [11].

Our study is aimed at describing the frequency, demographic features, clinical presentation, risk factors, etiologies, location and radiological characteristics, severity, management including surgical interventions, and outcome among young adults aged 15–45 years presenting with ICH to King Abdulaziz Medical City, MNGHA, Riyadh, which is one of the largest tertiary care centers in the kingdom.

## 2. Methods

### 2.1. Study Design and Settings

This was a retrospective cross-sectional, chart review study. It was approved by the IRB of King Abdullah International Medical Research Center with approval number NRC21R/134/04. It included young adults defined as age 15–45 years. In our center, pediatrics age is considered up to 14 years of age. The patients were further divided into three subgroups based on age; the youngest group comprised of patients between ages 15 and 25 years, the middle group comprised of patients aged between 26 and 35 years, and the oldest group comprised of patients aged between 36 and 45 years. All consecutive patients with the diagnosis of ICH who were admitted in the period from January 2016 to December 2020 at King Abdulaziz Medical City, MNGHA, Riyadh, Saudi Arabia, were included. The etiology of ICH was not known prior to including the patients in the study. The IRB waived the requirement for consent. Clinical, radiological, and outcome data were collected in a prespecified data collection form. The stroke severity was determined using National Institute of Health Stroke Scale (NIHSS) score, Glasgow Coma Scale (GCS), and ICH score at presentation, and outcome was measured using modified Rankin Scale (mRS) score.

### 2.2. Data Collection

The data on demographic, clinical, and radiological profile, as well as in-patient management, complications, and discharge outcomes were collected on a designed excel sheet from the electronic medical records (BestCare system). The demographic characteristics included age, gender, nationality, marital status, and occupational history. Clinical variables included vascular risk factors (comorbidities), smoking and substance use status, symptoms, signs, severity of stroke as measured by NIHSS score, GCS score, and ICH score at presentation. The NIHSS score of 0–5 is considered mild stroke, 6–15 is considered moderate stroke, 16–25 is considered severe stroke, and more than 25 score is considered very severe stroke. GCS of 3–4 is considered severe brain injury, 5–12 is considered moderate, and 13–15 is considered mild brain injury. The ICH score is a 7-point score ranging from 0 to 6 that takes into account GCS at presentation, age of patient, infratentorial origin of the ICH, volume of ICH (< 30 mL or more than 30 mL), and intraventricular extension. The ICH score of 4 is associated with 97% mortality at 30 days, whereas the ICH score of 5 is associated with 100% mortality at 30 days. Pertinent laboratory data including platelet count, international normalized ratio (INR), and any other coagulopathy were also recorded. The radiological variables included site of hemorrhage, size of hemorrhage, ICH score, underlying vascular abnormality, or other causes of hemorrhage. The management variables included conservative management, surgical intervention, type of surgical intervention, ICU admission, stroke unit admission, and target blood pressure. The outcome variables included in-hospital complications, discharge disposition, and discharge outcome based on mRS score, which is a 7-point score ranging from 0 to 6, with a score of 0–2 considered a good outcome, a score of 3–5 considered a poor outcome, and a score of 6 meaning death. The stroke outcome was further categorized into good outcome and poor outcome based on mRS. Long-term follow-up was not available on a large number of patients; hence, 3 months and 1 year outcomes were not available. The outcome data was mostly analyzed at the time of discharge.

### 2.3. Statistical Analysis

Data was collected in an Excel sheet and exported to SPSS for analysis. The data was analyzed using SPSS software Version 22. The descriptive statistics for all the patients were reported using frequency and percentages for categorical variables and mean ± standard deviation for continuous variables.

The Chi-square test, *T*-test, and/or ANOVA was used in the study for assessing the association of independent variables like age, gender, and vascular risk factors with stroke severity and outcome. The *p* value of < 0.05 was considered significant for all the tests applied.

## 3. Results

The study included 120 patients, with a mean age of 26.81 ± 7.42 years. More than half of the patient cohort, 67 (55.8%), belonged to the youngest age group. In contrast, the middle and oldest age groups constituted 38 (31.7%) and 15 (12.5%) of the patient population, respectively. There was an overall male predominance (91.7%), although the oldest age group exhibited a significantly higher proportion of females (33.3%; *p* = 0.006) (Table 1). The most common preexisting vascular risk factors among the patient cohort were the use of illicit drugs (18.7%) and smoking (17.2%). The distribution of risk factors showed no significant disparities across age groups, except for hypertension (5.8%), alcohol consumption (3.8%), and diabetes mellitus (2.5%). The incidence of hypertension and diabetes mellitus was significantly higher among the oldest age groups, whereas all instances of alcohol consumption were observed within the middle age group (Table 1).

Table 2 presents the clinical presentation of the patient cohort. The median GCS score among patients was 7, with an interquartile range of 3–11. Approximately 60% of the patients had an initial NIHSS score of 25 or higher, and the majority (96.6%) had an mRS score of 0–2 prior to presentation. There was no significant difference in total GCS score, NIHSS, and MRS between different age groups. Most (82.9%) patients demonstrated normal temperature and had no focal weakness (73.8%) or numbness (91.4%), while brainstem reflexes were absent among 12.4% of the patient cohort. The most common clinical feature among the patients was altered level of consciousness in 85.7%, loss of consciousness in 73.5%, and pupillary abnormalities in 48.3%. There was no significant difference in clinical presentation characteristics across age groups.

A large number of patients, 87 (72.5%) required intubation, of whom 33 (27.5%) ended up having tracheostomy tube placement. Pneumonia, seen in 60 (50.4%) patients, was the commonest complication, followed by brain edema in 47 (39.2%), seizures, urinary tract infection, and sepsis in 22 (18.3%) patients each. Other ICH-related and/or hospital-acquired complications are shown in Table 3. There was no significant difference in complication rate among different age groups.

The neuroimaging findings showed that the most frequent location of ICH among the patients was in the lobar region in 99 (83.2%), followed by the basal ganglia in 24 (20.2%), thalamus in 11 (9.2%), and cerebellum in 8 (6.7%). There were no disparities in the distribution of ICH locations across age groups, except for a significantly higher prevalence of midbrain hemorrhage in the middle age group (three out of four midbrain hemorrhages; *p* = 0.037). Associated radiological complications of ICH showed subarachnoid hemorrhage in 51 (42.9%) and subdural hemorrhage in 37 (31.1%). Other associated radiological abnormalities are described in Table 4, which did not differ between different age groups.

Table 5 describes the outcomes based on stroke severity, disability severity, and discharge disposition at the time of discharge. The mean length of hospital stay was 52 ± 109 days, with a median stay of 21 days. Stroke severity at discharge was not available for all patients. When available, the majority of the patients, 34 (60%), had NIHSS scores ranging from 0 to 5. Disability at discharge was available for most patients, showing good outcomes in 46 (43%) patients with mRS scores of 0–2, whereas 35 (32.7%) of patients had poor outcomes with mRS scores of 3–5. Then, 25 (20.8%) of patients died in the hospital. The mortality rate was notably higher among the oldest (40%) and middle (26.3%) age groups compared to the youngest age group (13.4%) (*p* = 0.043). The majority of patients, 82 (68.3%), were discharged to home, whereas 33 (27.5%) remained hospitalized at the 3-month period.

The characteristics of ICH are shown in Table 6, including the ICH score, ICH volume, and ventricular extension. There was no significant difference among various age groups in these parameters. Trauma emerged as the most common etiology of ICH in 101 (84.2%) patients, with a notable variation observed across age groups, particularly indicating a significantly higher proportion among the youngest age group (*p* < 0.001) as shown in Table 6. A large majority of patients, 92 (76.7%) did not undergo any surgical intervention. On the other hand, the most common surgical interventions among the patients were EVD insertion in 11 (9.2%) and decompressive craniotomy in nine (7.5%) patients.

Logistic regression analysis, as presented in Table 7, showed significantly higher odds of mortality among the oldest age group (OR 4.30, 95% CI 1.23–14.98, *p* = 0.022), higher ICH score (OR 3.37, 95% CI 1.86–6.09, *p* < 0.001), ICH volume > 30 mL (OR 16.40, 95% CI 5.35–50.26, *p* < 0.001), ventricular extension (OR 5.60, 95% CI 2.14–14.68, *p* < 0.001), nontraumatic ICH etiology (OR 3.59, 95% CI 1.26–10.26, *p* = 0.017), hyperthermia (OR 3.92, 95% CI 1.07–14.35, *p* = 0.039), brain edema (OR 5.85, 95% CI 2.21–15.53, *p* < 0.001), and hydrocephalus (OR 6.29, 95% CI 2.00–19.77, *p* = 0.002). On the other hand, the results showed significantly lower odds of death among patients with higher GCS scores at presentation (OR 0.71, 95% CI 0.59–0.85, *p* < 0.001).

## 4. Discussion

Only a few studies have been conducted on ICH in young adults in Saudi Arabia and internationally, and none have included both traumatic and nontraumatic ICH cases. This is the first study to investigate all types of ICH in young adults. A large amount of literature focuses on spontaneous ICH in younger patients, where most of the studies excluded traumatic ICH patients. We included patients with all etiologies of ICH and found trauma to be the commonest etiology, which may be one of the major reasons for differences from the previously published literature. Our results differed from those of most previously conducted studies in terms of demographics, as the mean age of our patients (26.81 years) was younger than what has been reported internationally (mean ages of 40.98 and 37.0 years in the Philippines and Taiwan, respectively, and a median age of 42.0 years in Finland) [5, 7, 8]. The younger mean age in our study might be explained by the inclusion of traumatic cases, as teenagers and young adults are more likely to engage in risky behaviors that can lead to physical trauma. Two previous studies focused on nontraumatic ICH in young adults had similar mean ages, but one included patients younger than 18 years old [10], and the other had a younger upper limit for age in their inclusion criteria (40-year-old) [9]. However, consistent with previous studies, our sample showed a significant male predominance [5, 7–10]. The disproportionately higher number of male patients in our cohort may also be because of a couple of additional reasons. Our center primarily caters to soldiers of the National Guard. Until recently, the soldiers were exclusively males. Additionally, females were not driving until recently and our data mostly involved the years when females were not driving.

Hypertension is traditionally considered to be the commonest etiology of spontaneous ICH, followed by cerebral amyloid angiopathy when all patients with ICH are included. However, in young adults, the etiology of ICH may vary. Many of the previous studies have excluded patients with traumatic ICH; however, our study included all patients with ICH in young adults, including traumatic ICH. In our study, the use of illicit drugs (18.7%) and smoking (17.2%) were the two potential contributors to ICH in young adults, while only 5.8% of our patients had hypertension as the cause of their ICH. This was not the case in the previously mentioned studies. The H-ATOMIC Classification System has been used to determine the etiology of ICH [12]. One such study that utilized the H-ATOMIC Classification System in patients under the age of 50 found hypertension to be a definitive cause of ICH in 36% of the patients, whereas any degree of hypertension was present in 82.7% of the cohort [12]. A recent study from Korea that looked at the potential contributors in nontraumatic spontaneous ICH found that hypertension, obesity, smoking, and cerebral small vessel disease were the predominant contributors to spontaneous ICH [13]. In the Philippines, hypertension was found to be the most common cause (74.1%), followed by alcohol abuse (37.8%) and smoking (30.3%) [5]. In Taiwan, hypertension was also the most common cause (37.6%), followed by rupture of AVMs (20%), while alcohol and illicit drug use each represented 1.8% [7]. In Finland, hypertension was the most common etiology (25%), but data on illicit drug use and alcohol consumption were lacking in almost half of the patients [8]. In Mexico, vascular malformations were found to be the most common cause of ICH in young adults (49%), followed by hypertension (11%), with 20% of their patients being smokers [9]. Similar results were reported previously in Saudi Arabia, where AVMs (23%) and hypertension (20%) were the most common causes [10]. Illicit drug use was higher in our study compared to the previously mentioned studies, which reported results ranging from 1.3% to 12.4% [5, 8–10]. This may be due to easier access to substances of abuse in the recent past. Encompassing all causes of ICH, including traumatic ICH in our study, likely contributed to the difference in risk factors or possible contributors to ICH.

Lobar hemorrhage (83.2%) was the most common location in our cohort, which is understandable given that it is the usual site of traumatic ICH and the low number of hypertensive bleeds in our cohort. However, the lobar site of ICH was also reported to be the most common site in two studies of ICH in young adults that excluded traumatic cases, with 41% and 55% in Taiwan and Mexico, respectively [7, 9]. Additionally, a lobar site of ICH was seen in 79% of the sample in a previous study of nontraumatic ICH in young adults in Saudi Arabia, when they excluded hypertension as a cause [10].

Almost 40% of our patients had good functional outcomes upon discharge (mRS scores of 0–2), which is slightly higher than what was reported in the Philippines and Finland (35.1% and 37.5%, respectively) [5, 8]. One of the possible reasons was that the overall ICH score was 2 in our cohort, which is associated with 26% mortality. An Indian study looking at the outcomes of ICH in patients aged below 50 years reported good outcomes in 58.1% of the patients at 90 days [14]. More than three-fourths of patients were males, and a little over half had a history of hypertension [14]. The mortality rate was 20.8%, which is within the range reported in previously mentioned studies (8%–35%) [5, 7–10]. A recent study on adults aged 18–45 with spontaneous ICH found an overall 30-day mortality rate of 15% [15]. This study also looked at the correlation of ICH score and mortality risk and found that the ICH scores overestimated the mortality risk in this age group. The actual mortality was much less when ICH scores of 1, 2, and 3 were applied to predict the mortality [15]. Additionally, most of our patients had supratentorial ICH with a volume of < 30 mL, which again are good prognostic factors in ICH.

Our study is limited by its retrospective design, the relatively small sample size and being a single center study, which may affect generalizability. Due to the retrospective nature, the missing data could not be accounted for and many assessments were made only for the patients on whom the actual data was available. In particular, the NIHSS score at admission and discharge, which describes the score severity, was missing in many patients. The disproportionate male-to-female ratio in our cohort limited gender-specific analyses. Future prospective studies with larger cohorts and longitudinal follow-up are warranted to validate our findings and explore evolving trends in ICH among young adults.

The predominance of traumatic ICH in our cohort necessitates targeted preventive strategies focused on injury prevention and education, particularly in younger age groups. Addressing modifiable risk factors such as substance use could potentially mitigate the incidence of traumatic ICH in this population.

## 5. Conclusions

Our study provides a comprehensive review of the clinical and radiological features, causes, and outcomes of ICH in young adults. We found a notable predominance of the male gender and identified trauma as the leading cause of ICH, highlighting important demographic and etiological patterns. Despite the lower occurrence of typical vascular risk factors, ICH leads to considerable morbidity and mortality. Large volume hemorrhages, ventricular extension, and relatively older age were poor prognostic factors.

## Figures and Tables

**Table 1:** Age, gender, and risk factors of patients in the cohort.

**Parameters**	**All (** **n** = 120**)**	**Youngest (15–25 years) (** **n** = 67**)**	**Middle (26–35 years) (** **n** = 38**)**	**Oldest (36–45 years) (** **n** = 15**)**	**p** ** value**
Age, in years (mean, SD)	26.81 (7.42)				
Gender					0.006⁣^∗^
Male	110 (91.7%)	65 (97%)	35 (92.1%)	10 (66.7%)	
Female	10 (8.3%)	2 (3%)	3 (7.9%)	5 (33.3%)	
Risk factors					
Use of illicit drugs	20 (18.7%)	13 (21.7%)	6 (18.2%)	1 (7.1%)	0.538
Smoking	16 (17.2%)	8 (15.1%)	4 (14.3%)	4 (33.3%)	0.327
Hypertension	7 (5.8%)	2 (3%)	1 (2.6%)	4 (26.7%)	0.007⁣^∗^
Drinking alcohol	4 (3.8%)	0 (0%)	4 (12.1%)	0 (0%)	0.020⁣^∗^
Diabetes mellitus	3 (2.5%)	0 (0%)	1 (2.6%)	2 (13.3%)	0.016⁣^∗^
Prior ischemic stroke	2 (1.7%)	1 (1.5%)	0 (0%)	1 (6.7%)	0.334
Dyslipidemia	2 (1.7%)	0 (0%)	1 (2.6%)	1 (6.7%)	0.095
Use of direct antithrombin	2 (1.7%)	1 (1.5%)	0 (0%)	1 (6.7%)	0.334
Ischemic heart disease	1 (0.8%)	0 (0%)	0 (0%)	1 (6.7%)	0.125
Thrombophilia	1 (0.8%)	1 (1.5%)	0 (0%)	0 (0%)	> 0.999
History of malignancy	1 (0.8%)	0 (0%)	0 (0%)	1 (6.7%)	0.125
Use of vitamin K antagonist	1 (0.8%)	0 (0%)	0 (0%)	1 (6.7%)	0.125
Use of heparin or LMWH	1 (0.8%)	0 (0%)	0 (0%)	1 (6.7%)	0.126
Using aspirin	1 (0.8%)	0 (0%)	0 (0%)	1 (6.7%)	0.125
Using clopidogrel	1 (0.8%)	0 (0%)	0 (0%)	1 (6.7%)	0.125

Abbreviations: LMWH, low molecular weight heparin; SD, standard deviation.

⁣^∗^Significant differences at the 0.05 level.

**Table 2:** Clinical presentation of patients in the cohort.

**Parameters**	**All (** **n** = 120^∗∗^**)**	**Youngest (15–25 years) (** **n** = 67**)**	**Middle (26–35 years) (** **n** = 38**)**	**Oldest (36–45 years) (** **n** = 15**)**	**p** ** value**⁣^∗^
Total GCS score (median, IQR)	7 (3–11)	8 (3–11)	7 (3–11)	7 (3–15)	0.691
Initial NIHSS^a^					0.680
0–5	19 (27.5%)	11 (28.2%)	5 (29.4%)	3 (23.1%)	
6–15	5 (7.2%)	3 (7.7%)	0 (0%)	2 (15.4%)	
16–24	4 (5.8%)	3 (7.7%)	0 (0%)	1 (7.7%)	
≥ 25	41 (59.4%)	22 (56.4%)	12 (70.6%)	7 (53.8%)	
MRS at admission					> 0.999
0–2	115 (96.6%)	63 (95.5%)	37 (97.4%)	15 (100%)	
3–5	4 (3.4%)	3 (4.5%)	1 (2.6%)	0 (0%)	
Weakness					0.785
Unilateral	17 (15.9%)	8 (14%)	5 (14.3%)	4 (26.7%)	
Bilateral	11 (10.3%)	7 (12.3%)	3 (8.6%)	1 (6.7%)	
Absent	79 (73.8%)	42 (73.7%)	27 (77.1%)	10 (66.7%)	
Numbness					0.657
Unilateral	3 (2.9%)	1 (1.8%)	1 (2.8%)	1 (7.1%)	
Bilateral	6 (5.7%)	3 (5.5%)	3 (8.3%)	0 (0%)	
Absent	96 (91.4%)	51 (92.7%)	32 (88.9%)	13 (92.9%)	
Temperature					0.096
Hypothermia	9 (7.7%)	7 (10.9%)	1 (2.6%)	1 (6.7%)	
Normothermia	97 (82.9%)	53 (82.8%)	34 (89.5%)	10 (66.7%)	
Hyperthermia	11 (9.4%)	4 (6.3%)	3 (7.9%)	4 (26.7%)	
Brainstem reflexes					0.870
Preserved	61 (54%)	33 (55%)	20 (52.6%)	8 (53.3%)	
Partially preserved	38 (33.6%)	20 (33.3%)	14 (36.8%)	4 (26.7%)	
Absent	14 (12.4%)	7 (11.7%)	4 (10.5%)	3 (20%)	
Altered level of consciousness	102 (85.7%)	56 (84.8%)	35 (92.1%)	11 (73.3%)	0.189
Loss of consciousness	86 (73.5%)	49 (75.4%)	26 (70.3%)	11 (73.3%)	0.873
Pupillary abnormalities	57 (48.3%)	28 (43.1%)	22 (57.9%)	7 (46.7%)	0.359
Focal weakness	21 (24.7%)	12 (25%)	5 (21.7%)	4 (28.6%)	0.887
Seizure/fit	22 (18.8%)	12 (18.2%)	6 (16.7%)	4 (26.7%)	0.660
Headache	14 (13.2%)	6 (10.5%)	4 (11.8%)	4 (26.7%)	0.276
Vomiting	13 (11.9%)	6 (9.4%)	4 (13.3%)	3 (20%)	0.443
Dysphagia	12 (15.6%)	9 (19.6%)	1 (5.6%)	2 (15.4%)	0.446
Loss of speech	11 (10.4%)	8 (14.3%)	2 (5.6%)	1 (7.1%)	0.456
Difficulty swallowing	8 (7.5%)	6 (10.3%)	1 (2.9%)	1 (7.1%)	0.390
Aphasia	7 (9.2%)	5 (11.6%)	2 (10%)	0 (0%)	0.658
Loss of vision/altered vision	6 (5.4%)	6 (9.8%)	0 (0%)	0 (0%)	0.103
Sudden vertigo	4 (3.5%)	1 (1.6%)	1 (2.6%)	2 (13.3%)	0.113
Fall	4 (3.5%)	2 (3.3%)	2 (5.3%)	0 (0%)	0.795
Sudden difficulty walking	3 (2.6%)	3 (4.9%)	0 (0%)	0 (0%)	0.532
Focal numbness	3 (4%)	2 (4.8%)	0 (0%)	1 (7.7%)	0.575
Double vision	1 (0.9%)	0 (0%)	1 (2.7%)	0 (0%)	0.451
Sudden balance problem	1 (0.9%)	1 (1.6%)	0 (0%)	0 (0%)	> 0.999
Dysarthria	1 (1.3%)	1 (2.3%)	0 (0%)	0 (0%)	> 0.999

Abbreviations: IQR, interquartile range; GCS, Glasgow Coma Scale; mRS, modified Rankin Scale; NIHSS, National Institute of Health Stroke Scale.

^a^Initial NIHSS score was documented in only 69 patients.

⁣^∗^Significant differences at 0.05 level. ⁣^∗∗^Some of the data points were not available in all patients.

**Table 3:** Complications among patients in the cohort.

**Parameters**	**All (** **n** = 120**)**	**Youngest (15–25 years) (** **n** = 67**)**	**Middle (26–35 years) (** **n** = 38**)**	**Oldest (36–45 years) (** **n** = 15**)**	**p** ** value**⁣^∗^
Intubation	87 (72.5%)	49 (73.1%)	29 (76.3%)	9 (60%)	0.476
Pneumonia	60 (50.4%)	31 (47%)	21 (55.3%)	8 (53.3%)	0.732
Brain edema	47 (39.2%)	20 (29.9%)	20 (52.6%)	7 (46.7%)	0.059
Tracheostomy tube placement	33 (27.5%)	21 (31.3%)	10 (26.3%)	2 (13.3%)	0.398
Seizures	22 (18.3%)	14 (20.9%)	5 (13.2%)	3 (20%)	0.667
Urinary tract infection	22 (18.3%)	13 (19.4%)	8 (21.1%)	1 (6.7%)	0.562
Sepsis	22 (18.3%)	11 (16.4%)	8 (21.1%)	3 (20%)	0.793
Gastrostomy tube	17 (14.2%)	12 (17.9%)	4 (10.5%)	1 (6.7%)	0.482
Decompressive surgery for brain edema	11 (9.2%)	5 (7.5%)	5 (13.2%)	1 (6.7%)	0.669
Recurrent hemorrhage	9 (7.5%)	6 (9%)	3 (7.9%)	0 (0%)	0.701
Deep vein thrombosis	9 (7.5%)	5 (7.5%)	4 (10.5%)	0 (0%)	0.621
Ischemic stroke	5 (4.2%)	2 (3%)	3 (7.9%)	0 (0%)	0.405
Pulmonary embolism	5 (4.2%)	2 (3%)	3 (7.9%)	0 (0%)	0.405
Depression	4 (3.5%)	2 (3.1%)	2 (5.7%)	0 (0%)	0.779
Fall	3 (2.8%)	2 (3.4%)	1 (3.1%)	0 (0%)	> 0.999
Myocardial infarction	2 (1.7%)	1 (1.5%)	0 (0%)	1 (6.7%)	0.334
Bedsores	2 (1.7%)	1 (1.5%)	1 (2.6%)	0 (0%)	> 0.999

⁣^∗^Significant differences at 0.05 level.

**Table 4:** ICH location by image of patients in the cohort.

**Parameters**	**All (** **n** = 120**)**	**Youngest (15–25** years**) (n** = 67**)**	**Middle (26–35** years**) (n** = 38**)**	**Oldest (36–45** years**) (n** = 15**)**	**p** ** value**
Neuro-imaging					
Lobar	99 (83.2%)	58 (86.6%)	32 (84.2%)	9 (64.3%)	0.132
Basal ganglia	24 (20.2%)	13 (19.4%)	10 (26.3%)	1 (7.1%)	0.334
Thalamus	11 (9.2%)	7 (10.4%)	3 (7.9%)	1 (7.1%)	0.902
Cerebellum	8 (6.7%)	3 (4.5%)	3 (7.9%)	2 (14.3%)	0.305
Midbrain	4 (3.4%)	0 (0%)	3 (7.9%)	1 (7.1%)	0.037⁣^∗^
Medulla	2 (1.7%)	0 (0%)	1 (2.6%)	1 (7.1%)	0.089
Pons	2 (1.7%)	0 (0%)	1 (2.6%)	1 (7.1%)	0.089
Radiological complication					
Subarachnoid hemorrhage	51 (42.9%)	27 (40.3%)	18 (47.4%)	6 (42.9%)	0.775
Subdural hemorrhage	37 (31.1%)	21 (31.3%)	14 (36.8%)	2 (14.3%)	0.330
Hydrocephalus	15 (12.6%)	9 (13.4%)	4 (10.5%)	2 (14.3%)	0.855
Epidural hemorrhage	10 (8.4%)	7 (10.4%)	1 (2.6%)	2 (14.3%)	0.227
Ventricular extension					0.601
Lateral or 3^rd^ ventricle	28 (23.5%)	17 (25.4%)	9 (23.7%)	2 (14.3%)	
4th and other ventricles	9 (7.6%)	3 (4.5%)	4 (10.5%)	2 (14.3%)	
4th ventricle only	4 (3.4%)	2 (3%)	1 (2.6%)	1 (7.1%)	
No ventricular extension	78 (65.5%)	45 (67.2%)	24 (63.2%)	9 (64.3%)	

⁣^∗^Significant differences at 0.05 level.

**Table 5:** Stroke severity, disability, and discharge disposition status of the patients.

**Parameters**	**All (** **n** = 120^∗∗^**)**	**Youngest (15–25 years) (** **n** = 67**)**	**Middle (26–35 years) (** **n** = 38**)**	**Oldest (36–45 years) (** **n** = 15**)**	**p** ** value**
NIHSS at discharge^a^					0.178
0–5	34 (59.6%)	20 (60.6%)	10 (71.4%)	4 (40%)	
6–15	4 (7%)	4 (12.1%)	0 (0%)	0 (0%)	
16–24	5 (8.8%)	4 (12.1%)	0 (0%)	1 (10%)	
≥ 25	14 (24.6%)	5 (15.2%)	4 (28.6%)	5 (50%)	
MRS at discharge					0.149
0–2	46 (43.3%)	30 (52.6%)	11 (31.4%)	5 (35.7%)	
3–5	35 (33.0%)	18 (31.6%)	14 (40%)	3 (21.4%)	
6	25 (23.6%)	9 (15.8%)	10 (28.6%)	6 (42.9%)	
Patient status					0.043⁣^∗^
Alive	95 (79.2%)	58 (86.6%)	28 (73.7%)	9 (60%)	
Dead	25 (20.8%)	9 (13.4%)	10 (26.3%)	6 (40%)	
Discharge to where					0.069
Discharged home	82 (68.3%)	52 (77.6%)	23 (60.5%)	7 (46.7%)	
Discharged to inpatient rehabilitation	1 (0.8%)	0 (0%)	0 (0%)	1 (6.7%)	
Discharged to long-term facility/nursing home	3 (2.5%)	2 (3%)	1 (2.6%)	0 (0%)	
Transferred to another hospital	9 (7.5%)	4 (6%)	4 (10.5%)	1 (6.7%)	
Discharged as dead	25 (20.8%)	9 (13.4%)	10 (26.3%)	6 (40%)	
Still admitted at 3 months	33 (27.5%)	22 (32.8%)	10 (26.3%)	1 (6.7%)	0.108

Abbreviations: mRS, modified Rankin Scale; NIHSS, National Institute of Health Stroke Scale.

^a^Initial NIHSS score was documented in only 57 patients.

⁣^∗^Significant differences at 0.05 level. ⁣^∗∗^Some of the data points were not available in all patients.

**Table 6:** ICH score and etiology of patients in the cohort.

**Parameters**	**All (** **n** = 120**)**	**Youngest (15–25 years) (** **n** = 67**)**	**Middle (26–35 years) (** **n** = 38**)**	**Oldest (36–45 years) (** **n** = 15**)**	**p** ** value**
ICH score (median, IQR)	2 (1–3)	2 (1–3)	2 (1–3)	1 (1–3)	0.912
ICH volume					0.471
< 30 mL	92 (81.4%)	52 (82.5%)	31 (83.8%)	9 (69.2%)	
> 30 mL	21 (18.6%)	11 (17.5%)	6 (16.2%)	4 (30.8%)	
IVH					0.859
Yes	41 (34.5.1%)	23 (34.8%)	14 (36.8%)	4 (26.7%)	
No	78 (65.5%)	43 (65.2%)	24 (63.2%)	11 (73.3%)	
ICH etiology					
Trauma	101 (84.2%)	61 (91%)	33 (86.8%)	7 (46.7%)	< 0.001⁣^∗^
Unknown	6 (5%)	2 (3%)	2 (5.3%)	2 (13.3%)	0.255
Vascular malformation	5 (4.2%)	1 (1.5%)	2 (5.3%)	2 (13.3%)	0.107
Hypertensive	4 (3.3%)	1 (1.5%)	1 (2.6%)	2 (13.3%)	0.100
Coagulopathy	3 (2.5%)	2 (3%)	0 (0%)	1 (6.7%)	0.227
Breast cancer metastases	1 (0.8%)	0 (0%)	0 (0%)	1 (6.7%)	0.125
Management					0.663
EVD insertion	11 (9.2%)	7 (10.4%)	2 (5.3%)	2 (13.3%)	
Craniotomy	9 (7.5%)	5 (7.5%)	2 (5.3%)	2 (13.3%)	
Craniotomy and EVD	3 (2.5%)	2 (3%)	1 (2.6%)	0 (0%)	
Craniectomy and EVD	3 (2.5%)	2 (3%)	1 (2.6%)	0 (0%)	
Craniectomy	2 (1.7%)	0 (0%)	1 (2.6%)	1 (6.7%)	
No surgical intervention	92 (76.7%)	51 (76.1%)	31 (81.6%)	10 (66.7%)	

Abbreviations: EVD, external ventricular drain; ICH, intracerebral hemorrhage; IQR, interquartile range; IVH, intraventricular hemorrhage.

⁣^∗^Significant differences at the 0.05 level.

**Table 7:** Logistic regression comparison on factors associated with patients' mortality in the cohort.

	**Odds ratio (OR)**	**95% CI for OR**	**p** ** value**
Oldest vs. youngest	4.30	(1.23–14.98)	0.022⁣^∗^
Total GCS score	0.71	(0.59–0.85)	< 0.001⁣^∗^
Total ICH score	3.37	(1.86–6.09)	< 0.001⁣^∗^
ICH volume (>30 mL vs. <30 mL)	16.40	(5.35–50.26)	< 0.001⁣^∗^
Ventricular extension (yes vs. no)	5.60	(2.14–14.68)	< 0.001⁣^∗^
ICH etiology (other vs. trauma)	3.59	(1.26–10.26)	0.017⁣^∗^
Hyperthermia vs. normothermia	3.92	(1.07–14.35)	0.039⁣^∗^
Brain edema (yes vs. no)	5.85	(2.21–15.53)	< 0.001⁣^∗^
Hydrocephalus (yes vs. no)	6.29	(2.00–19.77)	0.002⁣^∗^

Abbreviations: GCS, Glasgow Coma Scale; ICH, intracerebral hemorrhage.

⁣^∗^Significant differences at 0.05 level.

## Data Availability

All the data are available with the corresponding author and can be provided upon request after permission from the institutional review board.
